# Supplementary material for: Electrical and magnetic anisotropies in van der Waals multiferroic CuCrP2S6
Source: Nat Commun. 2023 Feb 15;14:840. doi: 10.1038/s41467-023-36512-1 (PMC9931707; doi:10.1038/s41467-023-36512-1)
Supplement: Supplementary file 1 — Supplementary Information [file 41467_2023_36512_MOESM1_ESM.pdf]

Supplementary Information

Electrical and Magnetic Anisotropies in van der Waals Multiferroic $CuCrP_2S_6$

Xiaolei Wang[1,*], Zixuan Shang[1], Chen Zhang[2], Jiaqian Kang[3], Tao Liu[4], Xueyun Wang[3,*], Siliang Chen[5], Haoliang Liu[5,*], Wei Tang[6], Yu-Jia Zeng[6,*], Jianfeng Guo[7], Zhihai Cheng[7], Lei Liu[2], Dong Pan[2], Shucheng Tong[2], Bo Wu[8], Yiyang Xie[8], Guangcheng Wang[1], Jinxiang Deng[1], Tianrui Zhai[1], Hui-Xiong Deng[2], Jiawang Hong[3] and Jianhua Zhao[2]

[1]*Department of Physics and Optoelectronic Engineering, Faculty of Science, Beijing University of Technology, Beijing 100124, China*

[2]*State Key Laboratory of Superlattices and Microstructures, Institute of Semiconductors, Chinese Academy of Sciences, Beijing 100083, China*

[3]*School of Aerospace Engineering, Beijing Institute of Technology, Beijing 100081, China*

[4]*National Engineering Research Center of Electromagnetic Radiation Control Materials, University of Electronic Science and Technology of China, Chengdu 610054, China*

[5]*Guangdong Provincial Key Laboratory of Semiconductor, Optoelectronic Materials and Intelligent Photonic Systems, School of Science, Harbin Institute of Technology (Shenzhen), Shenzhen 518055, China*

[6]*Key Laboratory of Optoelectronic Devices and Systems of Ministry of Education and Guangdong Province, College of Physics and Optoelectronic Engineering, Shenzhen University, Shenzhen 518060, China*

[7]*Department of Physics and Beijing Key Laboratory of Opto-electronic Functional Materials & Micro-nano Devices, Renmin University of China, Beijing 100872, China*

[8]*Key Laboratory of Optoelectronics Technology Ministry of Education, Beijing University of Technology, Beijing 100124, China*

*Correponding Authors Emails: Xiaolei Wang (xiaoleiwang@bjut.edu.cn), Xueyun Wang (xueyun@bit.edu.cn), Haoliang Liu (liuhaoliang@hit.edu.cn), Yu-Jia Zeng (yjzeng@szu.edu.cn)

Supplementary Figures:

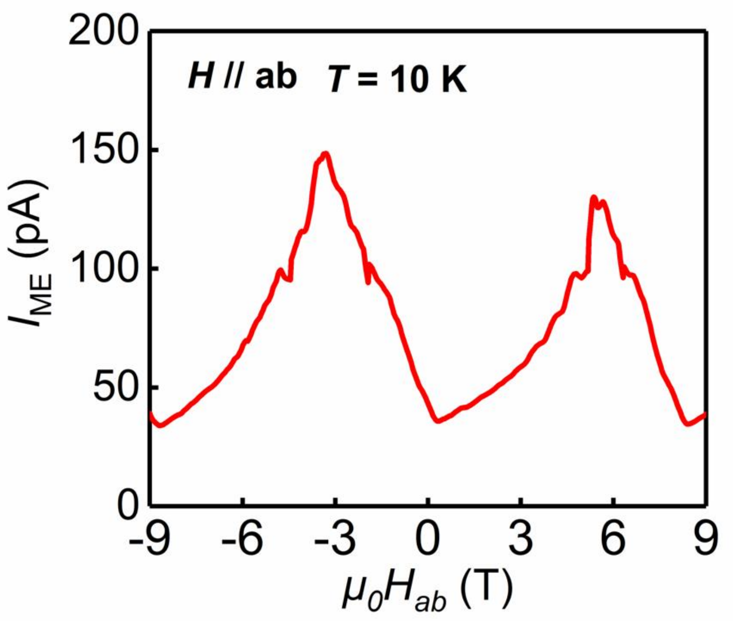

Supplementary Fig. 1 Magnetic field dependence of magnetoelectric current (I_{ME} ~ $\mu_0 H_{ab}$) measured at 10 K in the presence of *H* // ab-plane. An electric field E =2 kV cm^{-1} was applied along the *c*-axis. The I_{ME} curve exhibits two peaks at both positive and negative fields, which indicates the magnetoelectric coupling in CCPS.

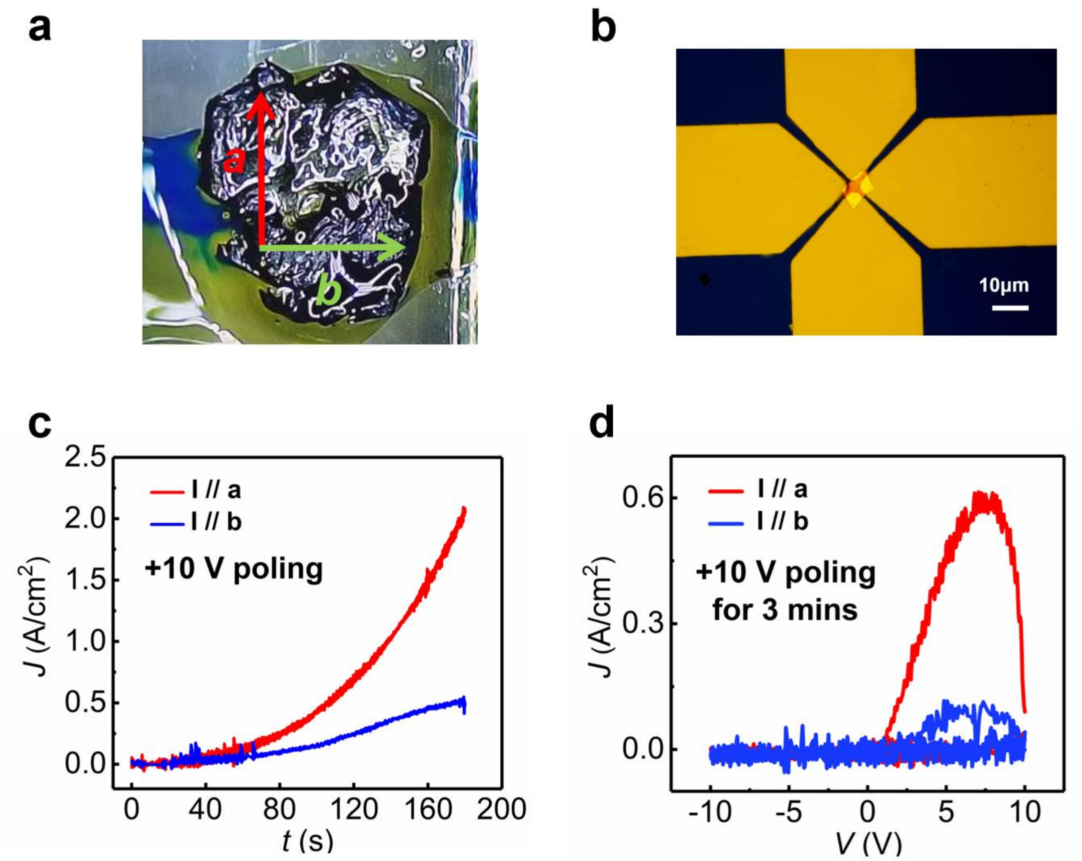

Supplementary Fig. 2 The electrical measurements of other device. a The photo of crystal bulk with the identified *a* and *b* axes for mechanical exfoliation and device fabrication. **b** The microscope picture of another CCPS device with film thickness ~55 nm. **c** The time dependence of current density along the *a* and *b* axes as setting the poling bias +10 V. **d** The rectifying current density *vs* sweeping voltage (*J*-*V*) characteristics along the *a* and *b* directions as applying + 10 V bias poling for 3 min.

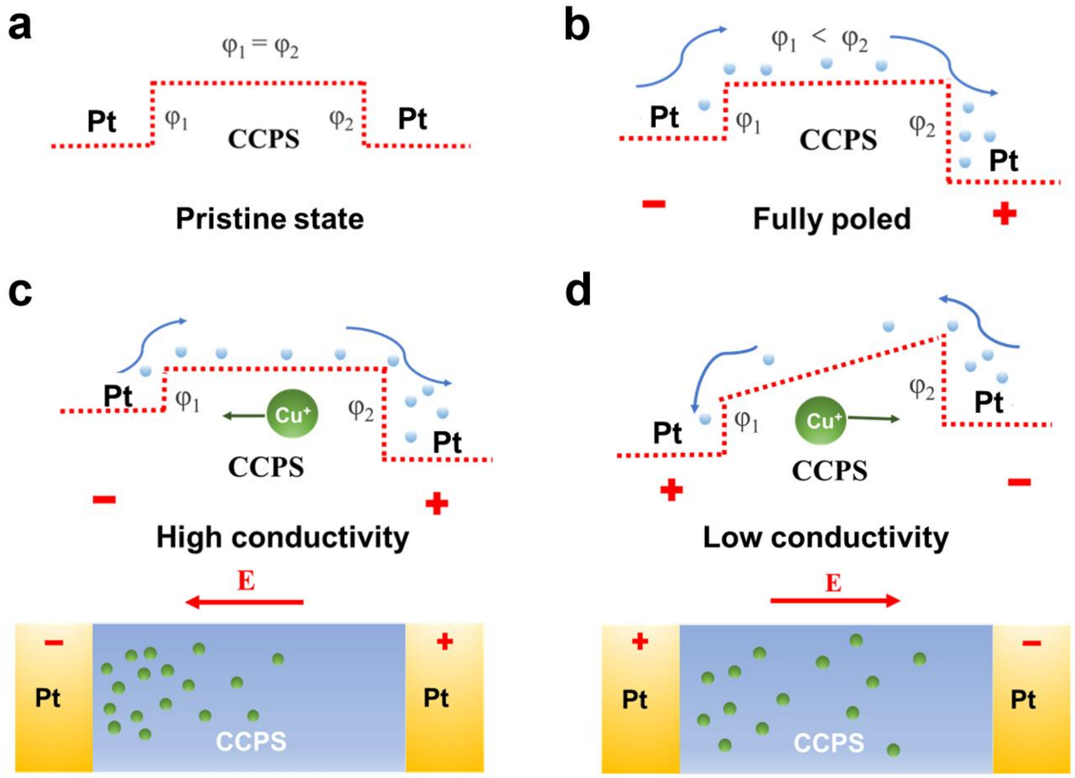

Supplementary Fig. 3 Schematics showing the ionic migration process in current rectification. a The band configuration of pristine state for Pt/CCPS/Pt device. **b** The evolution of two interfacial Schottky barriers under fully poled positive voltage. **c** Schottky barriers of high conductive state by positive voltage sweeping, and the corresponding model of Cu ions migration. **d** Schottky barriers of low conductive state by negative voltage sweeping, and the corresponding model of Cu ions migration.

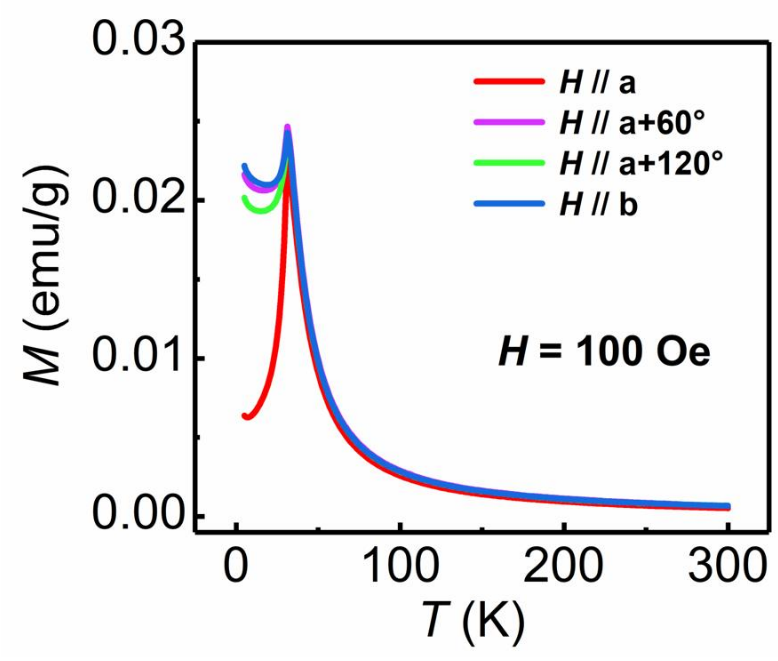

Supplementary Fig. 4 Temperature dependence of magnetic susceptibility (χ-T) along the *a*, *a*+60°, *a*+120° and *b* axes to exclude triple symmetry of the easy axes. The Cr ions form a honeycomb lattice in triangular networks of CCPS crystal, so the χ-T measurements with the 60° and 120° angle difference from the *a*-axis were obtained to demonstrate the uniaxial magnetic anisotropy, instead of triaxial anisotropy.

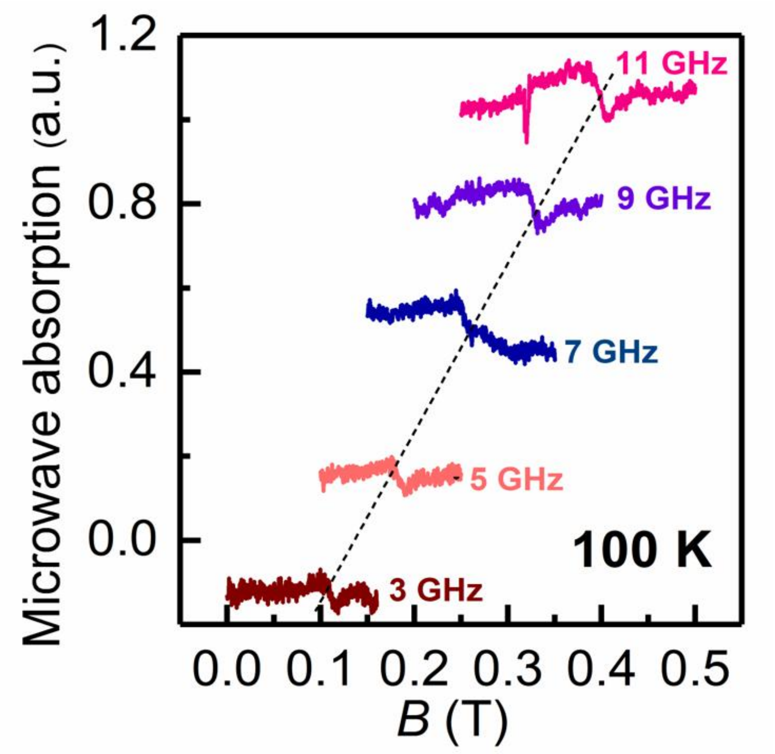

Supplementary Fig. 5 The magnetic field dependence of absorption spectra along the *a* axis with frequency 3~11 GHz at 100 K by antiferromagnetic resonance (AFMR) measurements. The position of single peak linearly shifts to high field as increasing the resonance frequency. It reveals an obvious electron paramagnetic resonance signal.

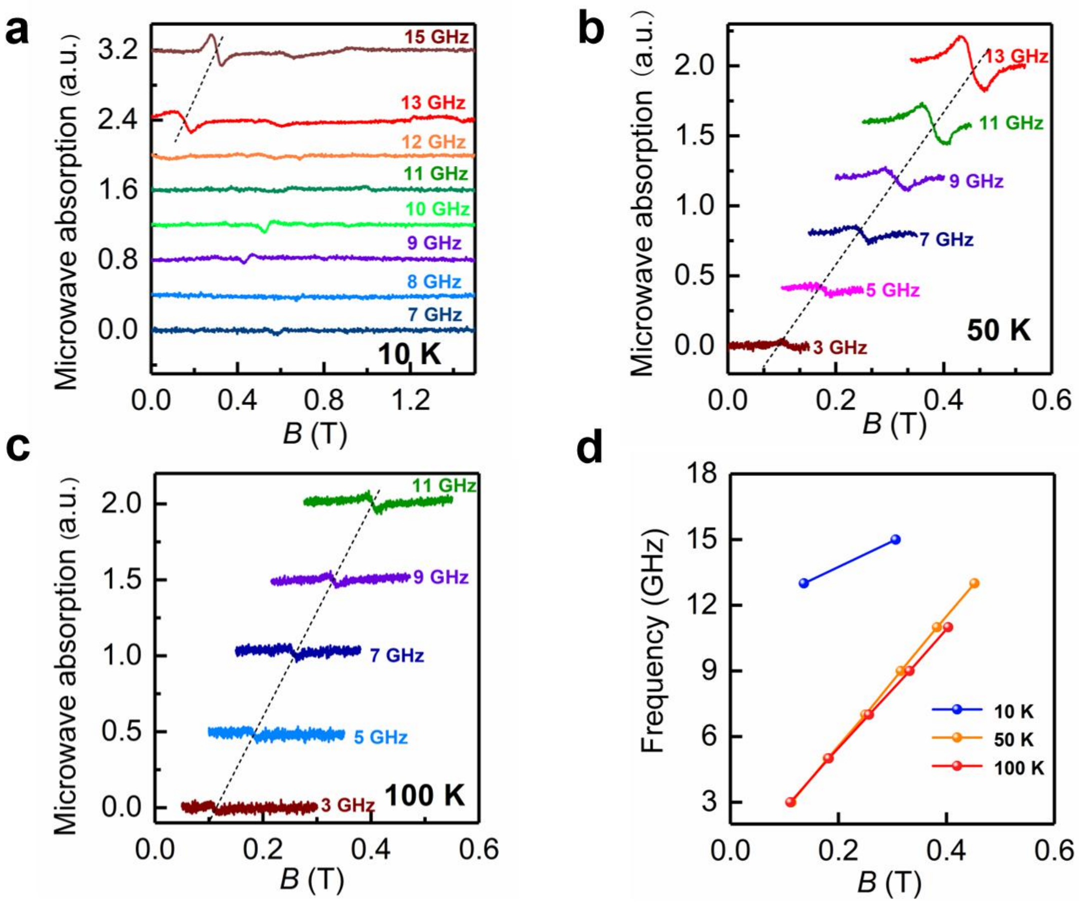

Supplementary Fig. 6 The resonance modes as *H* // b. The representative field dependence of absorption spectra with frequency 3~15 GHz at **a** 10 K, **b** 50 K and **c** 100 K by AFMR measurements. **d** The magnetic field dependence of resonance frequency at different temperatures.

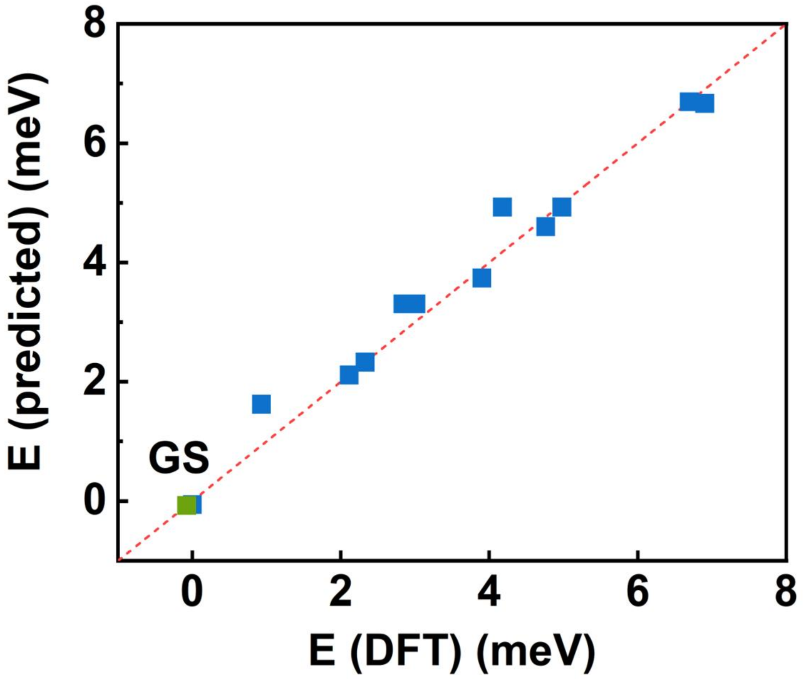

Supplementary Fig. 7 The the total energy fitting of 14 different spin structures. GS represents the magnetic ground state derived from the spinW package. It indicates that our DFT calculated total energies are highly consistent with predicted energies.

Supplementary Table 1. The 7 exchange parameters (*J*) for magnon calculations by DFT in our main manuscript, and *J*1~*J*7 are derived from Supplementary Fig. 7. The parameter A represents the anisotropy matrix.

Parameter	Energy (meV)
J1	-2.061
J2	-0.532
J3	-0.723
J4	-0.150
J5	-1.678
J6	0.124
J7	-0.086
A	$\begin{bmatrix} -4.651 & 0 & 0 \\ 0 & -5.809 & 0 \\ 0 & 0 & 0 \end{bmatrix}$ *1E-3
